# Bridging the Gap: Multidisciplinary Decision Making to Address Systemic Barriers in Cardiovascular Care

**DOI:** 10.1007/s11886-026-02375-3

**Published:** 2026-05-19

**Authors:** Erfan Tasdighi, Jerril Jacob, Kareena Patel, Anandita Kulkarni

**Affiliations:** 1 Department of Internal Medicine, Rutgers New Jersey Medical School, Newark, NJ USA; 2 Center for Cardiovascular Disease Prevention, Baylor Scott & White The Heart Hospital Baylor Plano, Texas, TX USA

**Keywords:** Cardiovascular disease, Health equity, Heart Team, Prevention, Social determinants of health, Structural barriers

## Abstract

**Purpose of Review:**

This review examines how social determinants of health contribute to inequities in cardiovascular outcomes and evaluates strategies for delivering equity-centered cardiovascular care across clinical, community, and policy settings.

**Recent Findings:**

Socioeconomic status, education, housing, food security, transportation, and insurance status significantly shape cardiovascular risk and care delivery. Multidisciplinary approaches integrating medical care with social support and community partnerships demonstrate promise in addressing these barriers. The Heart Team model, shared decision making, and quality improvement frameworks align clinical care with patient context. Evidence-based interventions including community-based programs, mobile health services, transportation assistance, and digital health tools improve cardiovascular access and outcomes among underserved populations.

**Summary:**

Addressing social and structural barriers is essential for reducing preventable cardiovascular morbidity and mortality. Future priorities include standardizing social risk data collection, expanding multidisciplinary care reimbursement, implementing equity-centered trial designs, and developing digital infrastructure supporting integrated care delivery.

## Introduction

**Fig. 1:**
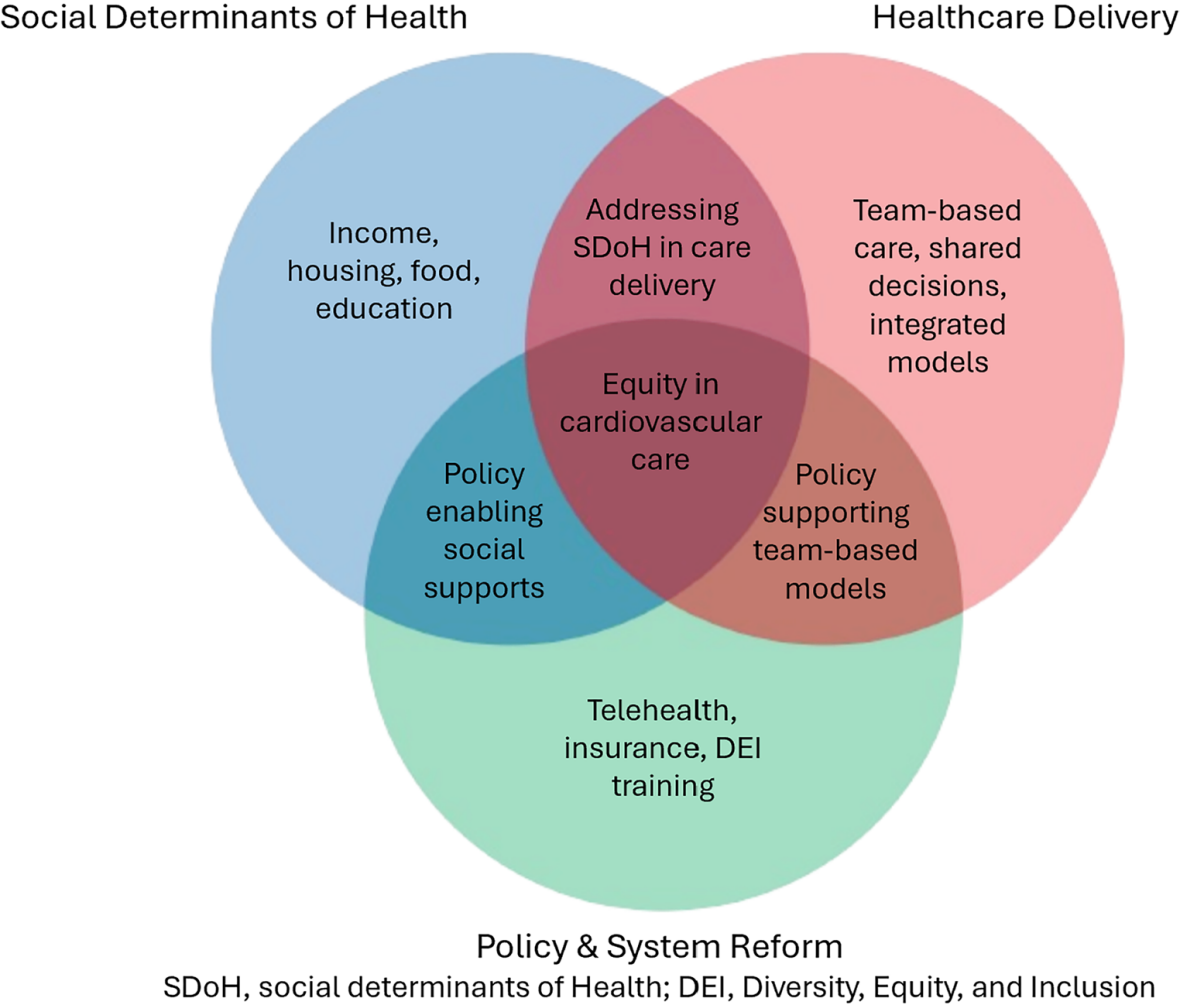
Drivers of equity in cardiovascular care

Cardiovascular disease (CVD) accounts for more than a quarter of all deaths in the U.S. in 2023,, according to the American Heart Association’s (AHA) 2026 Heart Disease and Stroke Statistics report [1]. As the largest contributor to the non-communicable disease (NCD) burden in 2021, accounting for an estimated 43.8 million deaths world-wide, CVD also leads in disability-adjusted life years (DALYs) lost, a metric capturing both premature death and years lived with disability [2, 3]. This immense health burden disproportionately affects marginalized populations across multiple dimensions. At the individual level, racial and ethnic minoritized groups bear a disproportionate risk. For instance, Black adults traditionally experience approximately a 30% higher rate of death due to CVD and a 45% higher rate of death due to stroke compared to non-Hispanic White adults [4]. Furthermore, vulnerable populations face socioeconomic and geographic disparities that further compound these inequities. Recent AHA and American College of Cardiology (ACC) scientific statements (2024–2025) reaffirm that Social Determinants of Health (SDoH) are primary drivers of cardiovascular (CV) risk, potentially more influential that biological variables [2, 5]. Studies demonstrate that cumulative disadvantage from multiple adverse SDoH confers a more than four-fold increase in atherosclerotic CVD risk [3]. Figure 1 demonstrates the drivers of CV care equity (Fig. 1).

The purpose of this review is to synthesize existing evidence to evaluate the roots of disparities in CVD healthcare, how multidisciplinary, equity-centered models have been applied to improve CV outcomes, and to identify practical implementation strategies that address persistent disparities in care delivery today. Using an integrative review methodology, this paper draws from diverse data sources, epidemiological studies, and evidence-based medicine to inform equity-focused, evidence-based strategies in CV care [6, 7]. This review examines existing team-based approaches, shared decision-making (SDM) frameworks, and the incorporation of community resources to address the interplay of medical and social challenges driving CV inequalities. By integrating clinical, social, and structural dimensions of care, the review aims to identify practical implementation strategies that reduce the growing burden of CV disparities globally through stronger alignment of clinical evidence, public health policy, and community-based engagement.

## Social Determinants of Cardiovascular Disparities

The interconnected forces, known as the SDoH, are central to understanding the persistence of CV disparities. SDoH encompasses the economic, social, environmental, and psychological conditions in which people live, work, and age, thereby profoundly influencing CVD and health outcomes [8]. Low socioeconomic status is strongly linked to a higher incidence and worse prognosis of CVD [9].

### Geographic and Racial Disparities

Geography is one of the most visible and consequential determinants of CV access and outcomes. Rural populations face longer travel distances to specialty centers, limited access to primary care, specialty cardiac services, and delayed emergency response times for acute cardiac events [4, 10]. However, geographic proximity alone does not fully explain disparities in CV care. Even after accounting for distance to available facilities, Black patients with acute myocardial infarction are less likely than White patients to be admitted to nearby high-quality hospitals or centers offering revascularization services [11], and decomposition analyses indicate that hospital geography accounts for only a portion of the White–Black gap in hospital quality for coronary heart disease, with nongeographic factors such as referral patterns and provider networks contributing substantially to these inequities [12]. These geographic barriers intersect with broader SDoH components that further increase CV risk [13].

### Insurance and Financial Barriers

About 1 in 12 people in the United States do not have health insurance [14]. Individuals without insurance are less likely to have a primary care provider and are additionally unable to afford healthcare services and medication costs. The financial barriers in CV care also extend beyond insurance, as people living in poverty are unable to afford healthy food and healthcare, which further increases CVD risk. Uninsured individuals with CVD experience delayed or forgone care and face higher rates of hospitalization and mortality compared to insured patients [15]. Even among those insured, high out-of-pocket costs contribute to medication nonadherence and worse health outcomes. For example, one analysis found that for a family with a median income of approximately $84,370 (2022 United States dollars), annual out-of-pocket medical expenses averaged $5,950 USD, in addition to approximately $6,100 USD in premiums, together consuming 14–15% of household income [16]. The most recent U.S. Census data show a 2023 median household income of $80,610 USD, underscoring how health insurance premiums and medical expenses continue to absorb a substantial share of income for middle-income families [17].

*The Healthy People 2030* initiative identifies economic stability as one of five key SdoH domains, alongside education access, healthcare access, neighborhood and built environment, and social and community context. Within the economic stability domain, the initiative targets factors such as employment, food insecurity, housing instability, and poverty, recognizing that steady income and access to basic resources are essential for maintaining good health, reducing disparities, and improving overall well-being [18].

### Educational Barriers and Health Literacy

Educational barriers and low health literacy further compound disparities by limiting understanding of disease processes, reducing treatment adherence, and increasing hospitalizations and mortality, particularly in heart failure, stroke, and diabetes [19]. The AHA identifies health literacy as a key determinant of CV outcomes, particularly among older adults and racial or ethnic minorities [20, 21]. Studies highlight that adults without a high school diploma have a 1.4 to 1.7 times higher lifetime risk of CV events and shorter longevity free of CVD compared with those who have higher educational attainment [22]. Lower education influences economic opportunity, health system navigation skills, and access to resources. Approximately 33% of CV patients have low health literacy, which correlates with nearly double the risk of mortality and a 35% higher risk of readmission [23]. Globally, CV risk factor control and outcomes decline with lower education levels, especially within low and middle-income countries, underscoring education as a critical social determinant to address in CVD prevention and care [24].

### Housing, Transportation, Food Insecurity

Housing instability, food insecurity, and inadequate transportation also increase CV risk. Food insecurity has a positive correlation with increased risk of hypertension, stroke, and heart failure [25]. Moreover, transportation barriers further complicate the challenge of effectively accessing preventative and emergent CV care. Reportedly, 5% of adults with atherosclerotic CVD reported delayed care due to a lack of transportation [26]. Additionally, studies show that adults experiencing housing insecurity are likely to have higher rates of hypertension, coronary artery disease, heart failure, and CV mortality [27].

### Physiological Stress

Addressing social and structural obstacles represents only part of the challenge. The physiological consequences of chronic socioeconomic adversity, including sustained inflammation, neuroendocrine and hormonal dysregulation, and oxidative stress, create biological pathways linking adverse social conditions such as poverty, housing instability, food insecurity, and discrimination to higher rates of CVD and mortality [28, 29].

## Limitations of Traditional Care Models

Historically, CV care has been provided through traditional, fragmented, physician-centered models that focus narrowly on individual behavior and are insufficient for addressing the complex interactions of medical, social, and structural factors that drive CVD [21, 22]. Current limitations to receiving CV care are nuanced, and many rural-based hospitals lack the resources to deliver timely and efficient emergency CV care; thus, the gaps in telehealth infrastructure, staffing, and referral networks only further exacerbate delays [30, 31]. Despite the multitude of targeted public health initiatives, CV mortality continues to rise disproportionately in rural regions, widening the rural-urban gap in health outcomes [32, 33]. These disparities intersect with social disadvantages such as poverty, food insecurity, and limited educational achievement [34, 35]. In addition, fragmented communication, limited interdisciplinary collaboration, and inequitable resource allocation continue to undermine progress, even in established healthcare systems. Understanding these structural and social determinants provides the foundation for implementing targeted interventions that improve CV equity. Despite advances in medical therapy and guideline-directed management, outcomes remain uneven. Advancing CV equity requires coordinated, multidisciplinary interventions that move beyond traditional risk factor management to integrate patient preferences, social context, and SDM frameworks [6].

## The Multidisciplinary Heart Team (HT) Framework

### Concept and Evolution

**Fig. 2:**
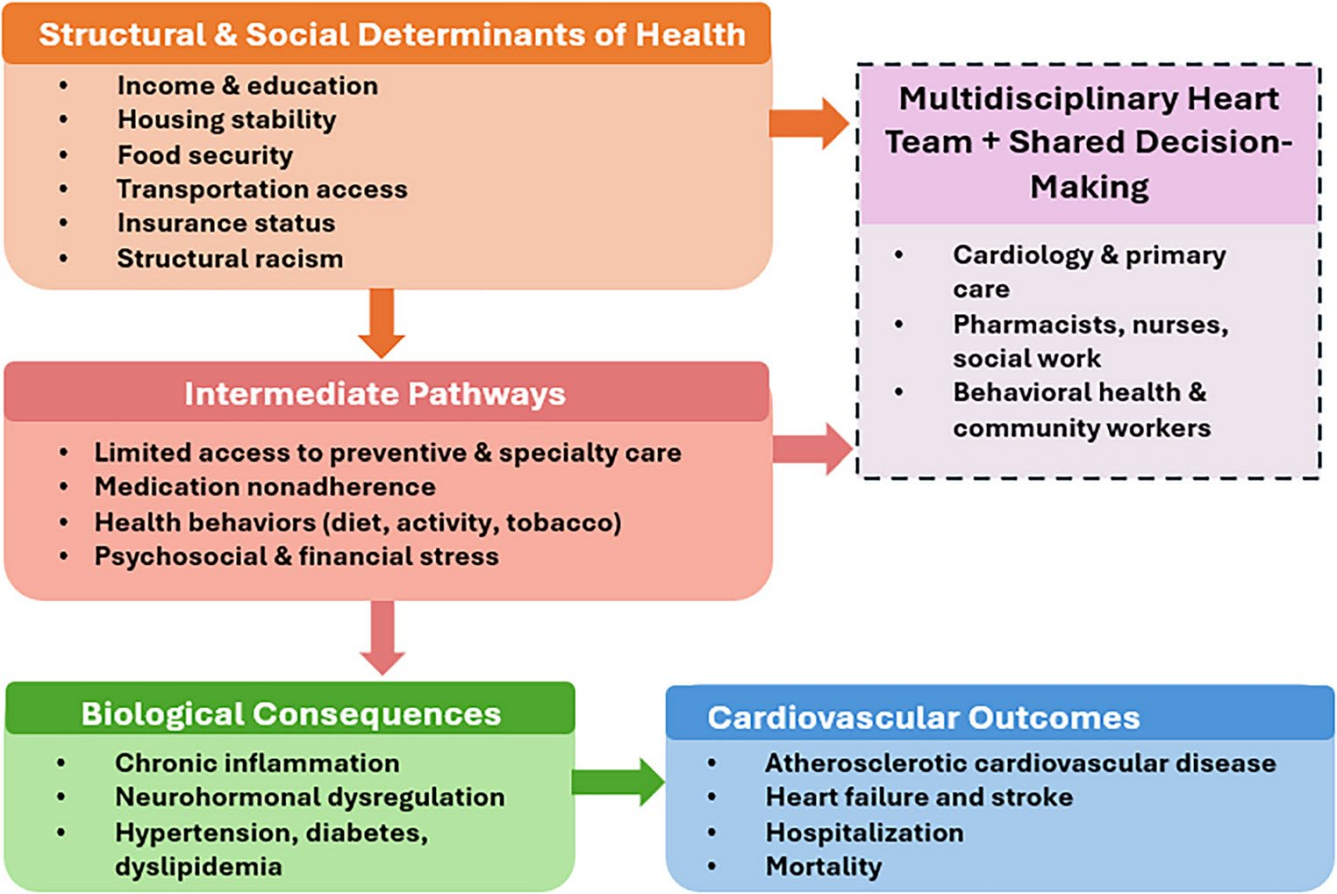
Integrated social-clinical pathway to cardiovascular outcomes

Building on these principles, effective CV equity depends on structured frameworks that operationalize multidisciplinary collaboration in clinical practice. Multidisciplinary decision-making relies on a structured framework that guides the development of individualized treatment plans [36]. In CV care, the central tenet of this framework is the Heart Team (HT) model, which facilitates complex clinical decisions through SDM [37]. This approach improves the quality of CV care and reduces disparities by accounting for the unique social contexts, specifically patients’ lived experiences of SDoH that influence health outcomes, thereby promoting health equity [38]. Establishing such a framework sets the foundation for understanding how multidisciplinary teams coordinate, communicate, and implement evidence-based strategies to address the multifaceted challenges of CVD (Fig. 2).

### Core Team Composition

Improving equity in CV care requires transforming policies, reimbursement structures, and the organization of care delivery itself [39]. The multidisciplinary HT model operationalizes equity by integrating diverse expertise to bridge the clinical and social dimensions of care [40]. The HT includes nurses, pharmacists, dietitians, social workers, and behavioral health professionals who address medical needs and social influences on health outcomes [41]. The HT has shown strong effectiveness in complex coronary and valvular disease management yet remains underutilized for addressing SDoH [41]. Principal members such as cardiologists, CV surgeons, primary care physicians, nurse practitioners, pharmacists, care coordinators, and nurses ensure care continuity across settings, manage therapies, and coordinate specialty services [42]. To address disparities, the HT incorporates professionals with expertise in social context, behavior change, and community engagement. Embedding an SDoH-informed SDM approach improves care quality and promotes equity in CV outcomes [39, 43].

Clinical pharmacists are essential members of the HT who optimize pharmacologic therapy, counsel patients, and align medication decisions with lifestyle and financial realities [40]. They manage treatments for hypertension, hyperlipidemia, diabetes, and heart failure while supporting adherence and preventive nutrition-based interventions [30]. Pharmacists identify untreated or undertreated risk factors, adjust therapy under collaborative agreements, monitor adverse effects or interactions, and assist patients facing cost or access barriers [31]. Integrating pharmacists into the SDoH-informed SDM model ensures medication management remains evidence-based, patient-centered, and equitable.

Social workers and case managers bridge clinical care with social and structural determinants of health. They assess barriers such as housing instability, food insecurity, employment, and transportation. They connect patients to social services, financial support, and community resources that strengthen treatment adherence and continuity of care. Their coordination during transitions from hospital to home improves medication reconciliation, follow-up, and multidisciplinary collaboration, reducing readmissions and improving outcomes [32]. At the systems level, these professionals identify institutional gaps, advocate for resource allocation, and collaborate with public health partners to address social risk factors [32].

Mental health professionals such as psychologists and behavioral therapists address depression, anxiety, and stress that worsen CV outcomes and hinder adherence. Integrated behavioral programs improve self-care and reduce hospitalizations [33]. Community health workers (CHWs) and patient navigators strengthen trust, reduce cultural and linguistic barriers, and assist with navigating healthcare systems [34]. Trials show that CHW-led programs sustain blood pressure control, improve appointment adherence, and increase preventive screening rates in Black, Latino, and South Asian populations [35]. Health educators and prevention specialists improve literacy and promote participation in SDM through culturally competent sessions tailored to patient needs [44].

### Quality Improvement

Quality improvement (QI) infrastructure supports the Heart Team by promoting coordination, accountability, and equity. Technology supports team coordination through electronic health records, real-time dashboards, and clinical decision support systems. QI frameworks that incorporate SDoH-sensitive metrics, such as race, zip code, and insurance type, identify disparities and measure progress [7]. Monitoring adherence to guideline-directed therapies, readmissions, and patient-reported outcomes strengthens accountability. Value-based care models reinforce equity-focused QI by linking reimbursement to health outcomes rather than service volume [45]. The HT and decision-making framework function as complementary components of equitable CV care. The HT brings multidisciplinary expertise to the bedside. The SDM framework ensures decisions reflect patient values and social context. Integrating these approaches creates a coordinated system of care that connects clinical precision with social responsiveness. This model advances the goals of *Healthy People 2030* by expanding access, improving quality, and promoting health equity across diverse populations [18].

## Integrating Social Determinants of Health into Cardiovascular Care

Translating SDoH evidence into practice requires embedding interventions within coordinated, multidisciplinary CV systems. The 2024 ACC/AHA report on SDoH defines standardized data elements and endorses patient-centered, community-integrated, and technology-enabled approaches to close access gaps [46].

The HT operationalizes these recommendations through a defined workflow: systematic SDoH screening at the point of care, structured case review at recurring interdisciplinary meetings, role-based task assignment, and closed-loop referral tracking through the electronic health record (EHR). Published examples demonstrate the model’s impact. The Grady Heart Failure Program [47], a safety-net HT in Atlanta, integrates cardiologists, advanced practice providers, pharmacists, social workers, and community health workers into a single clinic workflow and has been associated with higher rates of guideline-directed medical therapy and CDC recognition for advancing CV health equity [48]. The SMAC-HF trial similarly showed that a multidisciplinary group clinic visit intervention improved medication adherence and hospitalization-free survival in patients with social isolation and low health literacy, and the CDC’s WISEWOMAN program improved hypertension control among low-income women through pharmacist-led titration and team-based follow-up [49].

Within this workflow, food insecurity is screened using validated tools such as the Hunger Vital Sign, with positive screens triggering automated EHR referrals to dietitians and social workers who enroll patients in Supplemental Nutrition Assistance Program (SNAP), medically tailored meals, or produce prescription programs, interventions shown to lower blood pressure and HbA1c and reduce food insecurity [50–52].

Pharmacy and financial workflows operate on the same principle. When cost-related nonadherence is identified, the HT pharmacist initiates copay assistance, performs medication reconciliation, and coordinates insurance navigation through shared EHR notes [53, 54].

Housing instability is identified through standardized screening such as the Accountable Health Communities Health-Related Social Needs (HRSN) tool [55–57]. Positive screens activate the HT social worker, who coordinates formal healthcare, housing partnerships linking clinical sites with community service providers and housing agencies, an approach shown to improve continuity of care and reduce CV risk in under-resourced populations [58, 59].

The mechanism tying these workflows together is the closed-loop referral. EHR-integrated tools such as FindHelp and Unite Us allow any HT member to route a referral and receive confirmation when services are delivered, preventing identified needs from being lost between disciplines [60]. The mechanism tying these workflows together is the closed-loop referral. EHR-integrated tools such as FindHelp and Unite Us allow any HT member to route a referral and receive confirmation when services are delivered, preventing identified needs from being lost between disciplines [61, 62]. It is this integration that translates multidisciplinary staffing into measurable improvements in adherence, readmissions, and equitable outcomes [63, 64] (Table 1) [65–71].

**Table 1:** Evidence-based interventions addressing social determinants of health in cardiovascular care

Intervention Type	Setting	Targeted SDoH	Outcomes Improved
Medically Tailored Meals / SNAP [65]	Community, outpatient	Food insecurity	BP, HbA1c, diet quality
Transportation Assistance (rideshare, vouchers) [66]	Health systems	Transportation access	Appointment adherence, continuity
Pharmacy Assistance Programs [67]	Outpatient, Healthcare-based	Financial strain	Medication adherence
Community Health Workers [68]	Community-based	Navigation, trust	BP control, screening uptake
Mobile Screening Units [69]	Community settings	Geographic access	Early detection of HTN, diabetes
Telemedicine & Remote Monitoring [70]	Outpatient, rural	Geographic & access barriers	Follow-up rates, disease control
EHR-Integrated Social Referrals [71]	Health systems	Multi-domain SDoH	Closed-loop referrals, care coordination

*BP* blood pressure, *EHR* electronic health record, *HbA1c* hemoglobin A1c, *HTN* hypertension, *SDoH* social determinants of health, *SNAP* Supplemental Nutrition Assistance Program

## Technological and System-Level Innovations

### Telemedicine

Building on these equity-centered QI frameworks, the next frontier lies in leveraging digital infrastructure to operationalize them in daily practice. In modern CV care, digital tools have become integral to decision-making, enhancing coordination, patient engagement, and continuity across settings. Digital tools now support many of these decision-making processes. Telemedicine, remote monitoring, and electronic health records (EHRs) are integral in managing chronic CV conditions and facilitating timely interventions. Among underserved populations, telehealth expands access to subspecialty care. In contrast, EHR-based patient portals promote transparency and facilitate SDM, enabling patients to engage more actively in managing and tracking their care [72].

### Integrated EHR

Integrated EHR systems have a vital role in HT by allowing for seamless communication and coordination among providers in various settings. It allows for consolidation of clinical data, patient histories, and most importantly, social determinants information in an organized, unified record that can be readily accessible by the HT (cardiologist, nurses, pharmacist, social workers, behavioral health, etc.). This integration allows for SDM and care continuity, which includes screening for social needs such as housing instability or food insecurity [73]. In addition, patient portals linked to EHRs empower patients to take the lead on their health decisions by communicating with the care team and tracking their health status, which can also increase compliance to treatment plans. However, widespread EHR integration comes with its own set of challenges like interoperability gaps and data standardization barriers that require investment into health IT infrastructure that can fully realize promotion of team-based care [74].

### Decision Support

Clinical decision support systems (CDSS) integrated with EHRs provide the HT with evidence-based algorithms and alerts designed to improve care quality and outcomes [75]. These tools assist in CV risk assessment, guideline adherence, and personalized treatment planning by synthesizing patient data and recommending appropriate interventions, including those addressing social determinants of health. For example, CDSS can alert providers to uncontrolled hypertension (HTN), prompting cardiologists or multidisciplinary teams to initiate timely interventions. Studies have shown that CDSS improve screening rates and preventive care delivery, particularly when used consistently [75, 76].

### Data Analytics

While CDSS has a role in addressing SDoH in CV care, advanced data analytics plays a pivotal role in population health management. By aggregating and analyzing large amounts of data from EHRs and other sources, health organizations at the macro level can identify high-risk patient subgroups, monitor the prevalence of social determinants, and stratify risk to prioritize interventions appropriately. Data-driven dashboards allow care teams to track quality metrics such as hypertension control and hospitalization rates in real time, facilitating continuous improvement. For instance, it has been shown that measuring neighborhood deprivation can identify individuals at risk following cardiac hospitalization [77]. This supports proactive and preventive approaches needed to address the multifaceted needs of CV patients in diverse communities.

### Digital Health Equity and Infrastructure Gaps

The benefits of telemedicine and digital health innovations remain unevenly distributed across populations. Rural and low-income communities frequently lack broadband access, reliable cellular infrastructure, and digital literacy support, thereby limiting in value-based engagement with telehealth platforms and remote monitoring devices. Prior study showed that internet access rates were inversely associated with age-adjusted CVD mortality [78]. The practical application of these coordinated decision-making models depends on the development of digital systems and infrastructure that support timely communication, patient monitoring, and equitable access to care, ensuring that technology serves as a bridge rather than a barrier to equitable CV care.

## Evidence-Based Strategies for Barrier Mitigation

Bridging the gap between population-level analytics and individual care delivery demands scalable interventions that extend clinical reach into community settings that have a measurable impact across diverse populations. The following approaches discussed in this section complement the HT framework and emphasize implementation within real-world settings that reduce CV risk and narrow disparities.

### Mobile Screening

Expanding access to CV care for underserved populations increasingly relies on mobile health (mHealth) interventions. These tools leverage smartphones, wearable devices, and digital platforms to deliver preventive services, identify CV risk factors early, and promote sustained behavior change. Community-academic initiatives such as mobile screening units in local institutions like churches, barbershops, and supermarkets have improved screening rates for hypertension, diabetes, and dyslipidemia among vulnerable populations [42, 79]. The FAITH! App, a culturally tailored mobile intervention developed in partnership with African American churches, improved CV health behaviors by integrating spiritual, social, and behavioral support [43]. Digital tools that include goal tracking, text reminders, and real-time feedback enhance adherence to medications, diet, and physical activity across diverse populations [8, 80]. Aligning technology with community and cultural contexts builds trust and engagement, strengthening CV equity.

### Community-Based Care

Community-based care models bridge formal healthcare systems and patients’ lived environments by bringing services into neighborhoods, church halls, community centers, and homes. Person-centered CV delivery frameworks have demonstrated success when specialty services are integrated locally, reducing travel burdens and enhancing engagement [81]. In Rhode Island, a Community Health Team (CHT) program targeting high-risk patients reduced hospitalizations and inpatient cost burden [82]. These models often combine clinical and social support through embedding care managers, Clinical Health Workers, or social workers into primary care networks to address both medical needs and social determinants [82]. Community health workers who facilitated telehealth in low-resource settings have also shown efficacy in hypertension control when paired with home visits and clinician oversight [83]. Community-based structures capitalize on local trust and access, making them essential complements to centralized HT strategies.

### Transportation assistance

A meta-analysis of interventions targeting non-emergency transportation showed these programs reduced missed appointments by about 37% (pooled relative risk ~ 0.63) in clinics and hospitals [9]. Some hospital systems have responded with rideshare partnerships, travel vouchers, and lodging support; notably, over half of U.S. hospitals report programs addressing transportation barriers as part of their social needs strategy [25]. Embedding transportation assistance within HT-linked pathways through navigators or EHR referral prompts can help close the access-to-care gap and reinforce continuity in longitudinal CV management.

### Policy and Payment Models

Sustaining the HT model requires policy environments that promote interdisciplinary collaboration and long-term investment [84]. Programs such as CMS Innovation Models, the AHA’s “Target: BP,” and “Get With The Guidelines” align financial incentives with population-level outcomes [85]. Bundled payments, shared savings programs, and quality-based reimbursement frameworks encourage coordination across care settings [86]. Policy-level interventions, such as value-based payment reforms and clinical workflow integration of financial screening, co-pay assistance, insurance navigation, and benefits counseling, improve medication adherence and reduce preventable hospitalizations, thereby improving health equity outcomes in CV care [87].

## Challenges and Implementation Barriers

Efforts to integrate SDoH into CV care face various challenges across platforms of implementation, infrastructure, and policy landscapes. The most cited barriers are resource constraints and funding limitations [74]. Comprehensive implementation of SDoH-informed CV care requires elements such as policy reforms, alternative financing mechanisms, and stronger digital infrastructure.

### Funding

Reimbursement for non-physician providers, including clinical pharmacists, registered dietitians, and community health workers, remains inconsistent or altogether absent. This impedes the ability to build multidisciplinary teams essential for addressing the complex social and behavioral needs associated with CV risk [88]. Health systems serving vulnerable populations often lack sustainable financial models to support team-based services, especially those targeting upstream social drivers such as food insecurity, housing instability, and transportation deficits [89]. Broken funding streams and time-limited grant programs undermine long-term program viability [90, 91].

### Organization Culture

Additional limitations include persistent variations in organizational culture and readiness for change. While many institutions have adopted multidisciplinary care teams, genuine interprofessional collaboration is not always fully realized in practice. Structural barriers such as fragmented communication systems, inconsistent role clarity, and limited time for coordinated case discussions can hinder the effectiveness of these teams. Institutional culture is further reinforced by fee-for-service models that prioritize volume-based productivity rather than quality, equity, or prevention. Although there have been encouraging efforts to redesign workflows, redistribute responsibility, and promote community-based models, these initiatives are often constrained by limited leadership support, resource shortages, and workforce burnout [92–95].

### Regulatory & Policy

Regulatory and policy barriers continue to undermine the integration of comprehensive CV care [96, 97]. The current reimbursement frameworks under Medicaid and Medicare do not adequately incentivize coordination or the inclusion of behavioral health, care navigation, and communication-based social services, limiting the ability of health systems to address the broad determinants of CV risk and outcomes. National coverage for community-level interventions remains limited, despite strong evidence that primary care reforms such as task shifting and integrated team-based models improve hypertension and CVD outcomes. In addition, the lack of standardization through national guidelines for SDoH screening, risk stratification, or referral workflows in cardiology can lead to missed opportunities for preventive actions. Privacy and data-sharing laws, while designed to protect patients, can frequently block effective information exchange between clinical and social service providers required for coordinated care. The AHA, the National Lipid Association (NLA), and implementation scientists alike emphasize the need for standardized communication protocols, interprofessional training, and robust evaluation strategies to overcome these persistent hurdles [98].

### Technology and Interoperability

Disparate EHR systems, a lack of data interoperability, and an insufficient IT infrastructure impede coordinated care delivery. Many institutions lack the technical capacity to incorporate SDoH data into clinical workflows, risk prediction algorithms, or population health data. Community partners such as housing agencies or transportation services often operate outside healthcare platforms altogether, making cross-sector collaboration difficult to operationalize and evaluate. Without centralized platforms and shared governance models, multidisciplinary CV care anchored in social needs remains aspirational rather than functional [99].

### Measurement and Research Gaps

The full integration of SDoH into CV care continues to face persistent measurement and research challenges. Inconsistent data collection, absence of standardized outcome measures, and limited long-term funding hinder efforts to evaluate the true impact of SDoH-informed interventions [100]. These limitations restrict resource allocation, longitudinal impact assessment, and comparison of care models across settings [101]. They also constrain the ability to quantify progress in reducing disparities and to inform evidence-based policy development.

## Future Directions and Policy Implications

A concerted move toward hybrid effectiveness-implementation methodologies, equity-centered trial designs, and regular disaggregated data collection is necessary to fill these research gaps [102–104]. Achieving equity in CV care requires coordinated progress across research, policy, and technology. Isolated efforts will not close longstanding disparities; instead, health systems need integrated strategies that generate robust evidence, create supportive policy environments, and build digital infrastructure that operationalizes equity at scale. The following subsections outline the key research priorities, policy reforms, and technological innovations necessary to advance equitable CV care.

### Research Priorities

Future CV research must focus on how SDoH interventions can be effectively implemented and evaluated equitably across real-world settings. Methodologically, this calls for hybrid effectiveness, implementation trials within diverse health systems, with systematic and disaggregated reporting of outcomes by race, ethnicity, language, insurance type, and geographic region.

Recent AHA statements emphasize the need to integrate SDoH into genomic and precision medicine research [3]. This stems from data that suggests that chronic exposure to adverse social conditions can induce epigenetic modifications, such as DNA methylation, which biologically connect social stress and disadvantage, thereby perpetuating CV risk across generations [3]. To address these mechanisms, AHA and ACC call for longitudinal studies in ancestrally diverse populations and for precision prevention approaches that use SDoH-informed data to target interventions for those at the highest risk [2, 3, 5]. Therefore, CV research cannot fully support systems that improve outcomes and equity for socially at-risk populations until these changes are made.

### Policy Reforms

Integrating SDoH into CV care requires policies that promote not only equity-focused but also team-based care. Expanding reimbursement for multidisciplinary providers and aligning Medicaid and Medicare policies to support coordinated care are essential. Policy reforms like the Improving Seniors’ Timely Access to Care Act, which streamlines prior authorization in Medicare Advantage plans, and ongoing efforts to reform Medicare reimbursement models, can further reduce administrative burdens and improve timely access to care [105]. State grant programs and standardized SDoH screening can help improve preventive access and interdisciplinary collaboration. National initiatives like *Million Hearts® 2027* promote equity not only through insurance reforms but also encourage value-based models and culturally tailored interventions [106]. However, to maximize policy relevance, the field must also work toward standardized equity outcomes, such as time to guideline-directed therapy and quality of SDM, to promote meaningful comparisons and accountability across studies [107, 108].

### Technological Innovations

The 2025 ACC/AHA guidelines, through initiatives such as the PREVENT equations, call for the development of standardized SDoH data elements and their integration into EHRs and research to improve equity [39]. Advancing multidisciplinary CV care informed by SDoH depends on overcoming technological barriers. As discussed earlier, interoperability across EHR systems, centralized data platforms, and AI-based decision-support tools can enable real-time integration of both clinical and social data to guide shared decision-making among providers and patients [109–111]. Mobile health and telemedicine expand access to specialty care and health education in underserved areas. Therefore, building equitable digital infrastructure with user-friendly interfaces, digital literacy support, and robust data-governance frameworks is essential to ensure secure and inclusive collaboration across the Heart Team.

## Conclusion

CVD remains both the world’s leading cause of mortality and one of the most preventable non-communicable conditions when social, structural, and clinical inequities are addressed [112]. This review highlights how several barriers, such as food insecurity, housing instability, limited healthcare access, and structural racism, contribute to delayed diagnoses, suboptimal treatment, and inequitable CV outcomes [113, 114]. However, despite evidence supporting multidisciplinary, equity-centered models of care, their implementation remains inconsistent across regions and health systems [115].

Evidence-based strategies, including mobile health programs, community-based care delivery, transportation assistance, integrated behavioral health services, and telemedicine expansion, have identified meaningful improvements in access, patient engagement, and outcomes, particularly when adapted to local community needs [116].

Multidisciplinary HTs that incorporate principal roles of pharmacists, social workers, behavioral health professionals, and community health workers operationalize equity by aligning evidence-based care with patients’ social realities. Continued success, however, depends on long-term investment in workforce training, health IT infrastructure, and community partnerships, supported by policy and reimbursement reform.

Equity in CV care requires coordinated action at all levels of the health system. Clinicians must regularly screen for SDoH, incorporate social support services, and provide culturally responsive, person-centered care. Researchers need to design inclusive studies that break down data by race, ethnicity, and socioeconomic status to measure equity-focused outcomes and ensure interventions reflect diverse lived experiences. Additionally, policymakers and payers must maintain funding for multidisciplinary teams and prevention-oriented models that address upstream social and structural factors contributing to disease.

Achieving equitable CV health requires translating implementation science into practice, utilizing technology to address care gaps, and making equity a clear, measurable, and accountable goal in all aspects of CV care. This is not just a public health priority but also an ethical and moral necessity that shapes the future of CV medicine. Realizing equitable CV health will require translating implementation science into practice, leveraging technology to close care gaps, and embedding equity as a measurable, accountable goal in every aspect of CV care delivery. This is not only a public health priority but an ethical and moral imperative that defines the future of CV medicine.

## Key References

- Powell-Wiley, T.M., et al., Social Determinants of Cardiovascular Disease. Circulation Research, 2022. 130(5): p. 782–799.
  - ○ This paper establishes the mechanistic framework linking social determinants to cardiovascular outcomes through biological and behavioral pathways. It provides evidence-based strategies for addressing inequities at individual, community, and policy levels, making it foundational for understanding how social factors drive global CVD disparities.
- Palaniappan LP, et al. 2026 Heart Disease and Stroke Statistics: A Report of US and Global Data From the American Heart Associa­tion. Circulation. 2026;153(9):e275–906.
  - ○ This report provides current global epidemiological data quantifying CVD burden and disparities across racial, ethnic, socioeconomic, and geographic populations. It documents temporal trends showing where inequities are widening or narrowing, establishing the current scope of global cardiovascular health inequities.
- Brandt Eric, J., et al., Assessing and Addressing Social Determinants of Cardiovascular Health. JACC, 2023. 81(14): p. 1368–1385.
  - ○ This paper offers practical frameworks for screening social determinants in clinical settings and details evidence-based interventions for healthcare systems. It bridges theory and practice by providing actionable guidance for integrating SDOH assessment into routine cardiovascular care delivery.

## Data Availability

No data available.
